# Low temperature reduces potato wound formation by inhibiting phenylpropanoid metabolism and fatty acid biosynthesis

**DOI:** 10.3389/fpls.2022.1109953

**Published:** 2023-01-18

**Authors:** Jiadi Zhang, Jia Yao, Linli Mao, Qingpeng Li, Lixia Wang, Qing Lin

**Affiliations:** ^1^ College of Food Science and Engineering, Tianjin University of Science and Technology, Tianjin, China; ^2^ Key Laboratory of Agro-products Quality and Safety Control in Storage and Transport Process, Ministry of Agriculture and Rural Affairs/Institute of Food Science and Technology, Chinese Academy of Agricultural Sciences, Beijing, China; ^3^ School of Biomedicine, Beijing City University, Beijing, China

**Keywords:** potato tuber, temperature, healing, phenylpropanoid metabolism, fatty acid biosynthesis

## Abstract

**Introduction:**

Potato tubers have the healing capacity to prevent surface water transpiration and pathogen invasion after mechanical damage. Previous research has shown the inability to form healing periderm in potatoes under low temperatures, but the potential mechanism is still unclear.

**Methods:**

To explore the effects and mechanisms of low-temperature potato healing, wounded potatoes were stored at low temperature (4°C) and room temperature (22°C), respectively.

**Results:**

In this study, compared with 22°C healing, low temperature reduced the content of hydrogen peroxide, and the down-regulation of *StAMY23* inhibited the conversion of starch to sugar, alleviated the degradation of starch, and reduced the content of soluble sugars and sucrose. Meanwhile, inhibition of phenylalanine metabolism by suppression of *StPAL1* and *St4CL* expression reduced lignin accumulation. Low temperature also down-regulated the expression of *StKCS6*, *StFAOH*, *StGPAT5*, and *StPrx*, causing the lower deposition amount of suberin in wounds of potato tubers.

**Discussion:**

The above results suggested that low temperature led to less wound tissue deposition at the wound surfaces *via* suppressing phenylpropanoid metabolism and fatty acid biosynthesis in potato tubers.

## 1 Introduction

Potato (*Solanum tuberosum* L.) is an important staple vegetable in the human diet (Liu et al., 2014). It is extremely susceptible to mechanical damage during the harvest and transportation process, the wound surfaces of tubers establish a channel for infectious pathogens, preventing dehydration, browning, softening, unpleasant aroma, and taste deterioration (Saltveitm, 2016). However, potato tubers possess the healing ability, which usually takes about two weeks to form the functional barrier periderm on the wounds against pathogen invasion and prevent evaporation, to maintain the quality of tubers and greatly reduce post-harvest losses (Patil et al., 2012). Previous research has shown the optimal healing temperature of potato tubers is 22°C (HYODO, 1976). In response to healing, the wounded site synthesizes and deposits the biopolymer lignin and suberin, which is an induced defense response to plant damage and microbial attack (Vance et al., 1980).

In addition, sugar serves as an energy and carbon source for lignin and suberin synthesis in plants (Su et al., 2021). The evidence from published studies reveals that sucrose formed in the leaves of potato tubers during the ripening process can be transported to potato tubers and then transformed into starch for storage. Transformation of starch and sugar occurs in potato tubers during storage. Starch is decomposed into soluble sugars under the action of *α*-amylase (AMY) and *β*-amylase (BAM) (Borchert and Mcchesney, 1973). For instance, *AMY* and *BAMs* are involved in the starch degradation of kiwifruit during postharvest ripening (Hu et al., 2016; Zhang et al., 2018). Interference with *PtrBam1* in lemon inhibits starch degradation and soluble sugar content (Peng et al., 2014). In addition, ADP-glucose pyrophosphorylase (AGPase) is responsible for starch synthesis, and invertase (INV) is an irreversible enzyme in sucrose hydrolysis (Roitsch and Gonzalez, 2004). Up-regulated expression of *BAM* and *INV* has also been recently reported to be important for enhancing low-temperature resistance in potato tuber (Liu et al., 2021). Eventually, starch and sugar conversion generate glucose-6-phosphate (G-6-P) to enter the shikimic acid pathway (Li et al., 2018). Shikimic acid can be converted into erythrose for synthesizing phenylalanine (Tzin et al., 2013), an important precursor substance for potato wound formation (Li et al., 2018).

Phenylpropanoid metabolism is highly regulated by several stress factors and environmental stimulants (Zhang and Liu, 2015). Phenylalanine is an important substance involved in lignification (Ramamurthy et al., 2000). Phenylalanine ammonia-lyase (PAL) can catalyze L-phenylalanine to cinnamic acid and phenolics (Pendharkar and Nair, 1987). Members of *StPAL* families have different performances in potato tuber wounds, and the expression level of *StPAL1* is the most significant (Han et al., 2017). 4-coumarate: coenzyme A ligase (4CL) catalyzes the conversion of phenolic acid compounds to corresponding phenolic acid CoA, which can be reduced to corresponding aldehydes under the action of cinnamoyl CoA reductase. Bernards et al. (2000) found that 4CL activity was induced in the same way as PAL activity and it showed a similar trend to PAL during the healing of potato tubers.

However, the biosynthesis of suberin requires the coordinated deposition of two different biopolymers. One is suberin polyphenolic (SPP), which is deposited in the primary cell wall (Negrel et al., 1996). Another is the suberin polyaliphatic (SPA), which is involved in the biosynthesis of aliphatic monomers by fatty acid metabolism (Graca and Pereira, 2000a; Graca and Pereira, 2000b). Peroxidase (Prx) can participate in the peroxidative crosslinking of phenolics as part of SPP synthesis (Lulai and Neubauer, 2014). 3-ketoacyl CoA synthase (KCS) participates in chain-length fatty acids (≥ C28) to obtain ultra-long chain fatty acids. Silencing of *StKCS6* in potato periderm leads to reduced chain lengths of the suberin (Serra et al., 2009). Fatty acid *ω*-hydroxylase (FAOH) is a key enzyme for subcutaneous aliphatic biosynthesis in native periderm. Serra et al. (2009) applied RNAi technology and found that subcutaneous aliphatic in native periderm was reduced and ultra-structurally altered. Glycerol-3-phosphate acyltransferase (GPAT) encodes an acyl CoA-containing protein that catalyzes the key glycerol-based bridging between suberin SPP and SPA. It has been reported that in seeds, roots, and flowers, the reduction of very long-chain fatty acids due to the loss of *GPAT5* activity affects the binding of monomers in different ways (Beisson et al., 2007).

Overall, sugar metabolism, fatty acid metabolism, and phenylpropanoid metabolism are essential for the defense of potatoes during healing. The mechanism and expression patterns of the above genes in low-temperature treated potatoes during healing remain unclear. Therefore, in this study, potato tubers were wounded and then stored at 22°C and 4°C for up to 14 days. Physiological and biochemical indexes and the variation of metabolism pathway-related genes were analyzed. This study aimed to investigate the metabolic pathway of wound periderm formation in low-temperature treated potato tubers, to provide a theoretical basis for low temperature healing and to solve the problem of massive labor consumption after potato harvesting.

## 2 Materials and methods

### 2.1 Plant material, wound model system, and biochemical sampling

The potato ‘V7’ were purchased from Xinfadi, Beijing in October 2021. About 50 kg tubers were transported to the laboratory of Institute of Food Science and Technology, Chinese Academy of Agricultural Sciences on the same day. Tubers of uniform size and shape, free from diseases and without any visual defect were selected for the research. The tubers were carefully rinsed twice with distilled water, then immersed in 1.5% (v/v) sodium hypochlorite for 3 min to disinfect surfaces, and dried naturally. Afterward, three artificial wounds (approximately length × 1 cm, width × 1 cm, and depth × 0.5 cm) per tuber were created around the equator with a peeling knife. The wounded tubers were packed in 10-size polyethylene bags with holes (1- mm - diameter holes distributed every 3 cm). The tubers were stored at 22°C, and 4°C with 80% - 90% relative humidity in dark for healing. Wound tissues (3 mm) were collected from the wounded site using a knife after 0, 3, 5, 7, and 14 d of storage. Three biological replicates per treatment were used in all experiments. The samples were stored at -80°C.

### 2.2 Observation of wound surface and determination of weight loss rate

The weight of each potato was recorded at each sampling point. Eventually following calculation formula: Weight loss (%) = (m_0_ - m)/m_0_ ×100%, where ‘m_0_’ represents the wounded potato weight on the first day, ‘m’ represents the weight of the wounded potato at each sampling point of both control and treatments. Then take photos to record the wounded surface.

### 2.3 Observation of lignin and suberin accumulation

The vertical wound surface of the tuber was cut into slices (about thickness × 0.2 - 0.3 mm, width × 1 cm) with a blade. The slices were immediately rinsed with distilled water to remove starch granules and then immersed in 1% (w/v) phloroglucinol solution for 1.5 min on a glass slide with a few drops of concentrated hydrochloric acid. After 5 min, place the slide under a microscope (10×) to observe the lignin. A slice was cut in the vertical wound surface of the tuber, leaving the slices at 0.05% toluidine blue for 45 min. The residues were removed by washing with distilled water and 75% alcohol two times, respectively. Afterward, rinse twice with 95% alcohol. Finally, the tissue was stained with 1% neutral red III for 1 - 2 min and washed with distilled water and 75% alcohol. The SPA was observed by a (10×) microscope.

### 2.4 Determination of lignin and H_2_O_2_ content

Lignin content was determined by Lignin Content Detection Kit (Solarbio, China). Take 3 mg of dry sample and add the reagents according to the instructions. After thorough mixing, the acetylation reaction was carried out in a water bath at 80°C for 40 min, centrifuged at 8000 g for 10 min, and the supernatant was added with glacial acetic acid. The test was conducted in a 96-well UV plate according to the manufacturer’s instructions strictly, and absorbance was read at 280 nm using a microplate reader. H_2_O_2_ content was determined by H_2_O_2_ Content Detection Kit (Solarbio, China). Weigh 0.05 g of fresh sample in 500 µL acetone ice bath homogenization, centrifuged 8000 g for 10 min at 4°C, and the reagents was added into 250 µL supernatant according to the instructions. The precipitate was dissolved after mixing and centrifuged at 4000 g for 10 min, and rested for 5 min. Absorbance values were measured at 415 nm using a 96-well plate according to the manufacturer’s instructions.

### 2.5 Determination of starch, soluble sugar, and sucrose content

The starch, soluble sugar, and sucrose content were measured following the methods described by He (1985), and the absorbance value was measured at 620 nm. Take 0.01 g dry sample, and add 80% ethanol, 80°C water bath. After cooling, centrifuge to take the supernatant, and repeat the above operation. The supernatant was used to measure the sugar content and the precipitation was used to measure the starch. The supernatant was added 30% KOH and 300 µL anthrone reagent to measure the content of sucrose. The supernatant was added 300 µL anthrone reagent to measure the content of soluble sugar. The precipitation was diluted, add 9.2 mol L^-1^ perchloric acids, and centrifuged to obtain the supernatant. Add 300 µL anthrone reagent to the supernatant to measure starch content.

### 2.6 RNA extraction and cDNA synthesis

The total RNA of the potato tuber was extracted using RNA prep Pure Plant Kit (TIANGEN Biotech, China) according to the manufacturer’s instructions. The RNA integrity was determined using agarose gel electrophoresis (1% agarose gel, 0.5 × TAE, 100 V, 20 min), and purity was established at an absorbance of an OD_260_/OD_280_ ratio. Then the RNA was reverse transcribed to cDNA by iScript™ cDNA Synthesis Kit (TRANSGEN, China). All reactions for each cDNA sample were carried out in triplicate.

### 2.7 Real-time quantitative PCR

Primers used in the research were included in **Supplementary Table 1**. The *EF1α* gene was used as an internal control and was shown to be stable under the conditions used. The gene expression of *Prx*, *PAL1* (Lulai and Neubauer, 2014), *4CL*, *KCS6*, *FAOH*, *GPAT5* (Lulai and Neubauer, 2014), *AMY23*, *BAM1*, *AGPase* and *INV1* (Xie et al., 2018) were analyzed. cDNA was generated using Power SYBR Green PCR Master Mix kit (Applied Biosystems) and real-time quantitative PCR was conducted on ABI 7500 instrument (Applied Biosystems). The real-time PCR conditions were as follows: a pre-incubation at 94°C for the 30 s, then amplification of 40 cycles of 94°C for 5 s, subsequently 30 s at 60°C, 15 s at 95°C, 34 s at 60°C, and final extension step for 30 s at 95°C. The relative quantification of each gene was calculated using the 2^− ΔCT^ method and compared with the gene at the initial time.

### 2.8 Statistical analysis

All data were analyzed with Microsoft Excel 2019 software, which was used to calculate the mean value and standard deviation. The pictures were prepared using origin 8.5 software (Microcal Software Inc., Northampton, MA, USA). Differences between the control and treated tuber were assessed with a significant level of *p* < 0.05.

## 3 Results

### 3.1 Changes in physiological and biochemical indexes of potatoes under different temperature healing

**Figure 1:**
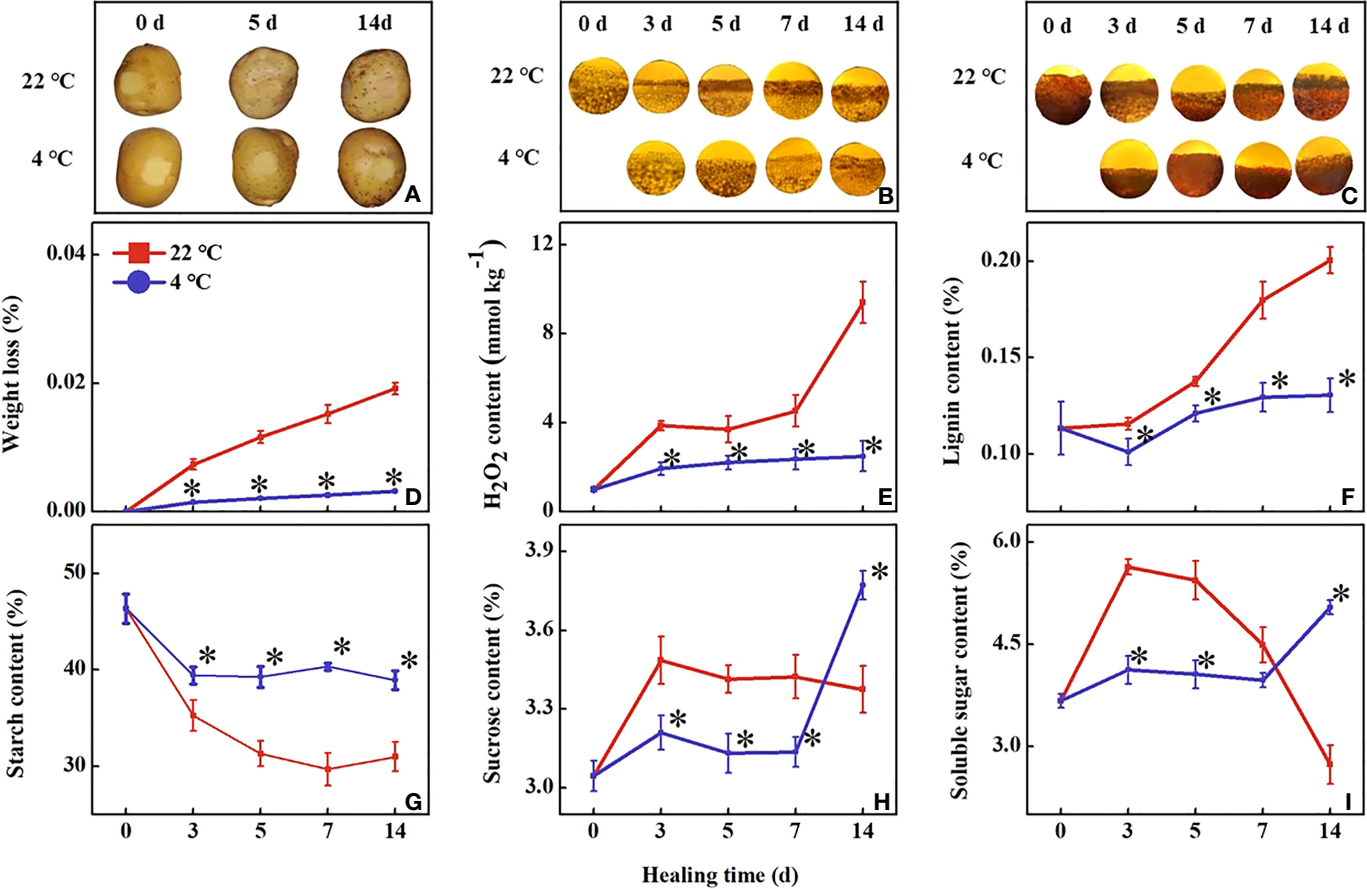
Effect of different temperatures on **(A)** observation of wound periderm surface, **(B)** lignin deposition, **(C)** suberin deposition, **(D)** weight loss, **(E)** lignin content, **(F)** H2O2 content, **(G)** starch content, **(H)** soluble sugar content and **(I)** sucrose content at the wound sites of potato tubers during healing. The symbol (*) indicates significant differences among different treatments at p < 0.05.

During healing, the wound periderm formation in tubers of all treatments continued to increase. The low-temperature treated wound periderm was reduced to a brighter color than the control within 14 days (**Figure 1A**). The accumulation of lignin has been increasing during healing in all tubers. The lignin accumulation was slower in the tubers under low-temperature treatment than in the control (**Figure 1B**). Similarly, suberin accumulation of all tubers also increased continuously during healing, however, the accumulation in the low-temperature treated tubers was significantly slower than that in the control (**Figure 1C**). The cell layers thickness of lignin and suberin increased in all tubers, and low-temperature treated tubers were lower than the control within 14 days. It indicated that the low temperature inhibited wound periderm formation and the accumulation of lignin and suberin at the wound sites.

During healing, the weight loss of all wounded tubers continued to increase. The weight loss rate of the low-temperature treated tubers became significantly slower than the control within 14 d (**Figure 1D**). The H_2_O_2_ content in the low-temperature treatment was significantly lower than the control, the control tubers first increased, then maintained, and finally increased to a high level. At 3 d and 14 d of healing, the H_2_O_2_ contents in the control tubers were 2-fold and 3.7-fold higher than that of the low-temperature treated tubers, respectively (**Figure 1E**). The lignin content in the control tubers showed a rapid increase during healing. The lignin content in the low-temperature treated tubers was significantly lower than that of the control during healing (**Figure 1F**).

During healing, starch content in all tubers decreased continuously. The content of starch in low-temperature treated tuber was significantly higher than in the control (**Figure 1G**). The content of soluble sugar initially increased and then rapidly decreased in the control tuber while that in the low-temperature treated tubers increased at 7 d of healing (**Figure 1H**). The sucrose content variation trend was similar to the soluble sugar content during healing (**Figure 1H**).

### 3.2 Changes of sugar metabolism in potato tubers during healing

**Figure 2:**
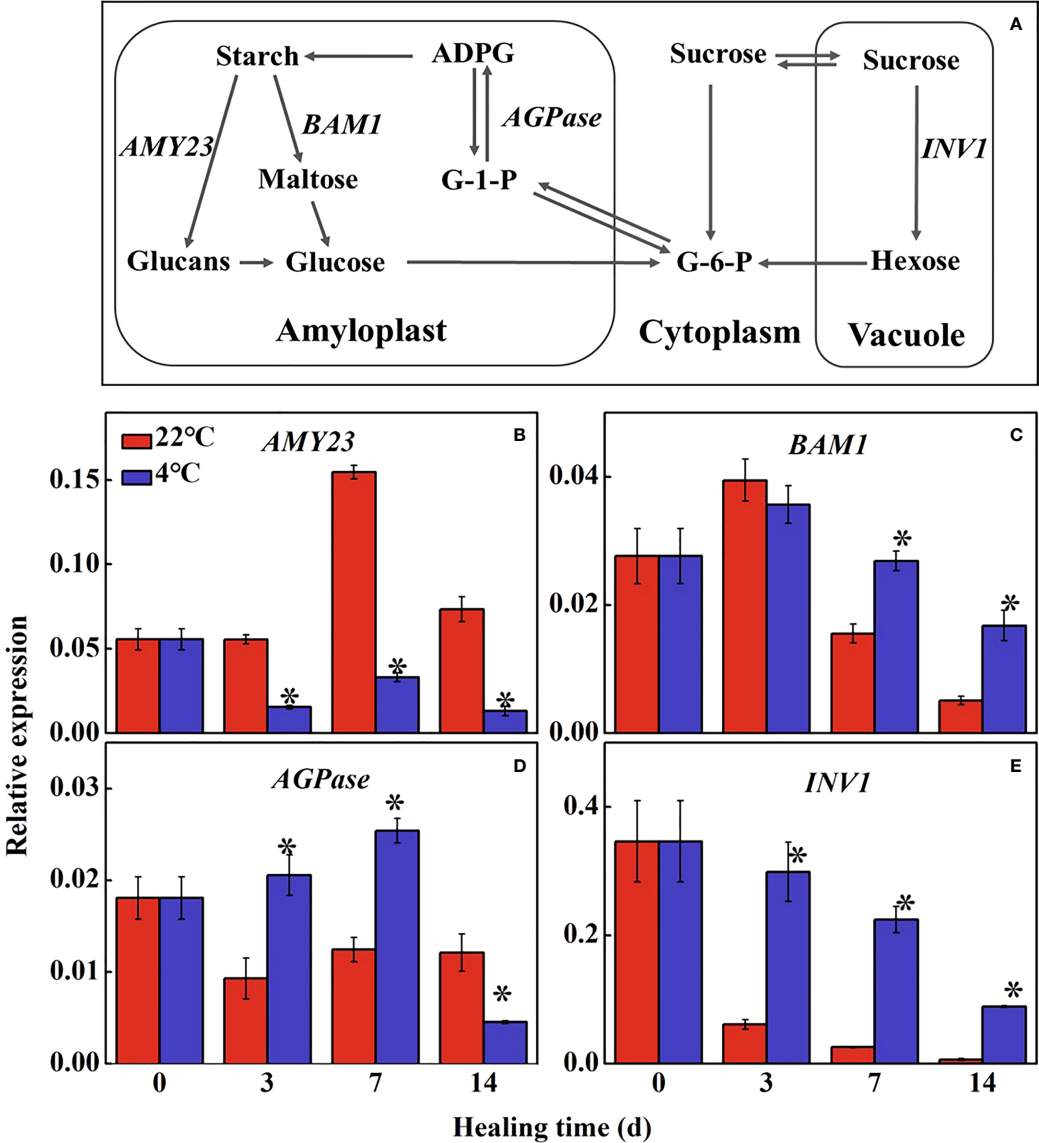
Figure 2. **(A)** The pathway of starch and sugar metabolism and effect of different temperatures on the expression of **(B)** alpha-amylase 23 (AMY23), **(C)** beta-amylase 1 (BAM1), **(D)** ADP-glucose pyrophosphorylase (AGPase) and **(E)** vacuolar1 (INV1) at the wound sites of potato tubers during healing. Starch is hydrolyzed into glucose under the alpha-amylase (AMY) and beta-amylase (BAM); G-6-P and glucose-1-phosphate (G-1-P) are interconverted to adenosine diphosphate glucose (ADPG) by ADP-glucose pyrophosphorylase (AGPase) for further starch synthesis. Sucrose is catalyzed into hexose (glucose and fructose) by sucrose invertase (INV). The symbol (*) indicates significant differences among different treatments at p < 0.05.

Starch and sugars are important energy supply in tuber during healing (**Figure 2A**). The expression of *StAMY23* in the control increased to the maximum level at 7 d of healing and was significantly higher than that in the low temperature-treated tubers (**Figure 2B**). Expression of *StBAM1* in the treated tubers was 1.7-fold and 3.2-fold higher than that in the control at 7 d and 14 d of healing, respectively (**Figure 2C**). The expression of *StAGPase* reached the maximum expression level at 7 d of healing, and in treated tubers was 2.0-fold higher than that in the control (**Figure 2D**). *StINV1* also decreased gradually during healing. The expression of *StINV1* was significantly higher in the low-temperature treated tubers than that in the control (**Figure 2E**). The results showed that low-temperature could inhibit the conversion of starch to sugar in the wound periderm of tubers, thus suppressing energy and precursor substance supply for healing.

### 3.3 Changes of fatty acid biosynthesis in potato tubers during healing

**Figure 3:**
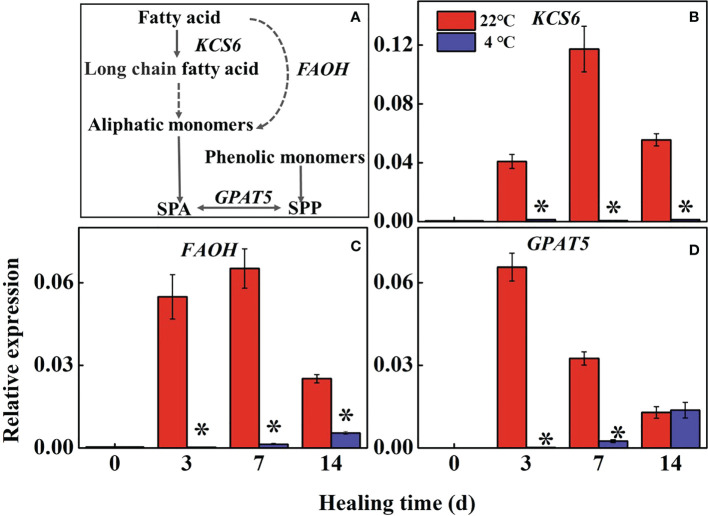
**(A)** The pathway of fatty acid metabolism and effects of different temperatures on the expression of **(B)** 3-*Ketoacyl-CoA synthase (KCS6)*, **(C)** *fatty acid-hydroxylase (FAOH)*, and **(D)** *glycerol-3-phosphate acyltransferase (GPAT5)* at the wound sites of potato tubers during healing. The symbol (*) indicates significant differences among different treatments at p < 0.05.

Fatty acid metabolism reflects the accumulation of suberin in the wound periderm of potato tubers during healing (**Figure 3A**). The expression of *StKCS6* and *StFAOH* in the controls were significantly higher than the low-temperature treated tubers during healing, while those in low-temperature treated tubers maintained at a low level (**Figures 3B, C**). The expression of *StGPAT5* rapidly increased in the control at 3 d and 7 d of healing compared to the low-temperature treated tubers (**Figure 3D**). The result indicates that low temperature could inhibit the expression of *StKCS6*, *StFAOH*, and *StGPAT5* to suppress suberin formation during healing.

### 3.4 Changes of phenylpropanoid metabolism in potato tubers during healing

**Figure 4:**
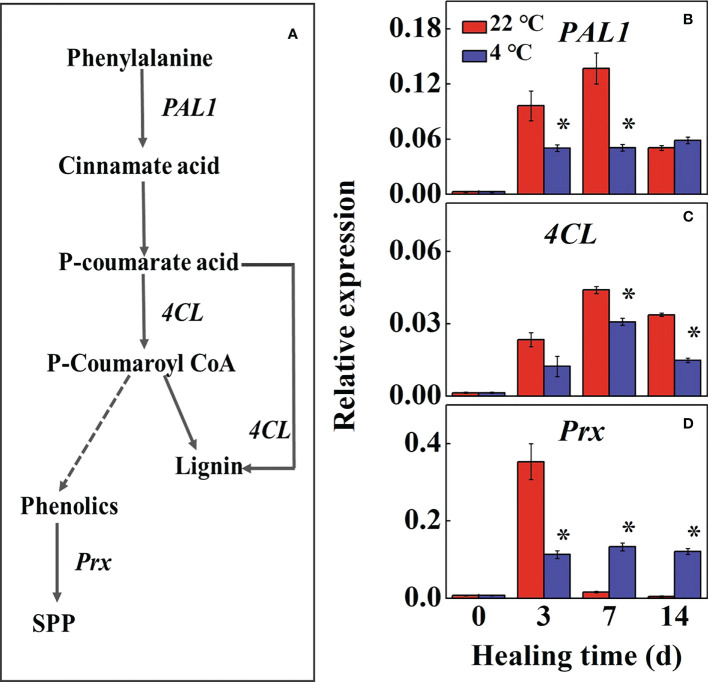
**(A)** The pathway of phenylpropane metabolic and effects of different temperatures on the expression of **(B)** Phenylalanine ammonia-lyase (PAL1), **(C)** 4-coumarate-CoA (4CL) and **(D)** anionic peroxidase (Prx) at the wound sites of potato tubers during healing. The symbol (*) indicates significant differences among different treatments at p < 0.05.

The phenylpropane metabolic pathway in the wound tissue reflects the ability of tuber healing (**Figure 4A**). During healing, the expression of *StPAL1* and *St4CL* increased first and then decreased in control compared to the low-temperature treated tubers, the maximum expression reached 2.7-fold and 1.4-fold at 7 d, respectively (**Figures 4B, C**). The expression of *StPAL1* and *St4CL* in low-temperature treatments were significantly lower than that of the controls, while the *StPAL1* expression in the low-temperature treatment was stable during healing. The expression of *StPrx* in the control was rapidly up-regulated at 3 d, and the low-temperature treatment was significantly lower than the control. However, the control tubers declined to near basal levels of expression at 7 d during healing (**Figure 4D**). The results suggested that low temperature mainly inhibited *StPAL1* and *St4CL* expressions to suppress lignin formation. Meanwhile, *StPrx* suppressed further conversion of phenolics to SPP.

### 3.5 Pattern of metabolic pathways in potato tubers during healing

**Figure 5:**
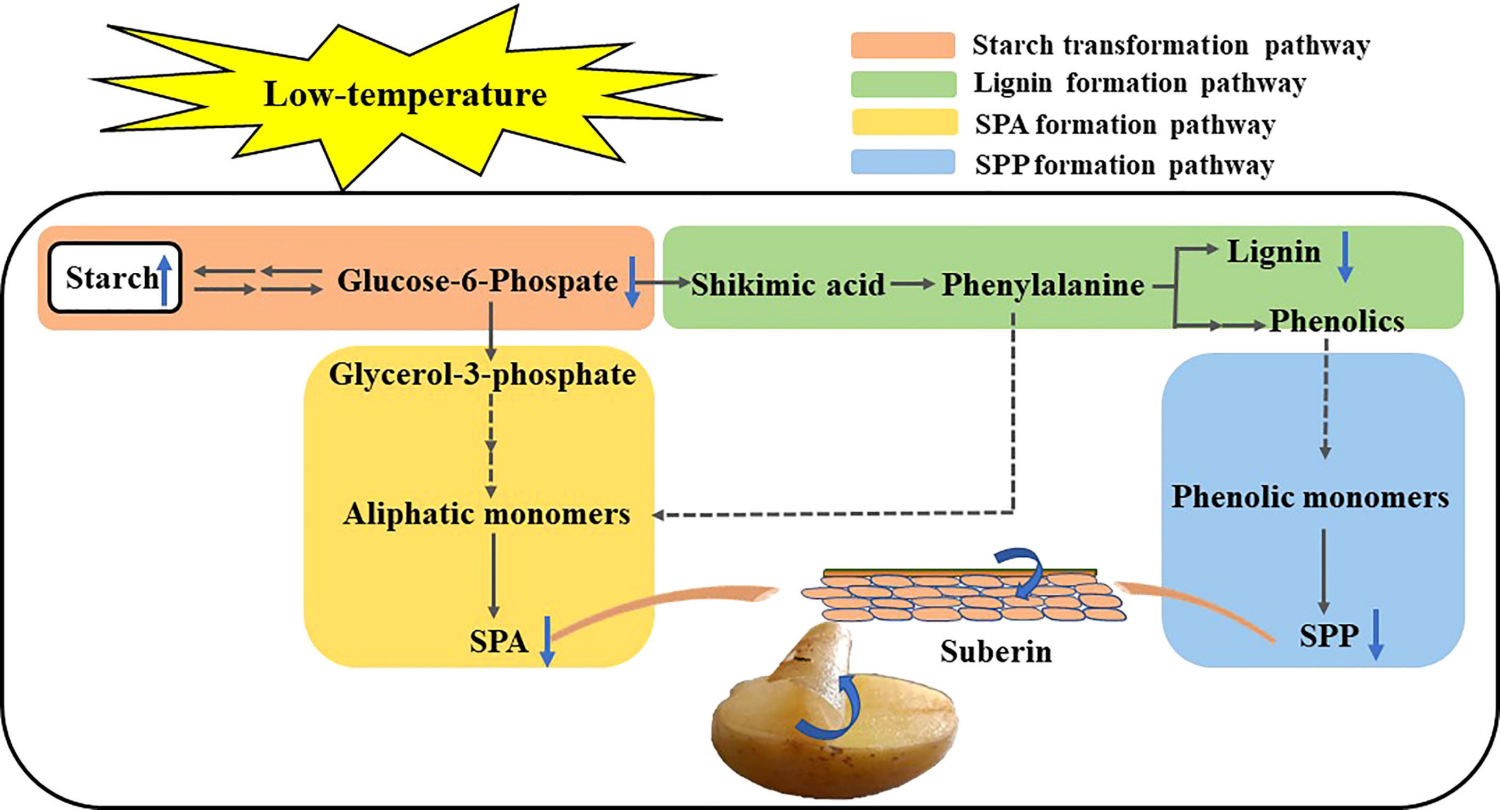
Pattern of metabolic pathways involved in potato wound healing.

Low temperature inhibits the conversion of starch to sugar in the wound periderm of tubers, thus suppressing energy and precursor substance supply for healing. Starch is converted to G-6-P *via* sugar metabolism. On the one hand, G-6-P enters the pathway of pentose phosphate and glycolysis, and its intermediate substances are converted to shikimic acid. Shikimic acid is converted into phenylalanine through phosphorylation and other reactions to enter the phenylpropane metabolic pathway. The phenylalanine is mainly catalyzed by phenolic acids, aldehydes, and alcohols, synthesizing less lignin and SPP. On the other hand, G-6-P is converted to glycerol-3-phosphate *via* the glycolytic pathway, which further forms super long chain fatty acids, ultimately, form small amounts of SPA that are deposited in the wound site (**Figure 5**).

## 5 Discussion

It is widely studied that potatoes at room temperature can accelerate the formation of wound periderm during healing (Jiang et al., 2019). Therefore, temperature has a very important role in the healing of potato tubers. The wound-healing response of fresh-cut apples and pineapples was promoted at 4°C, increasing H_2_O_2_ production and enhancing the activities of PAL and peroxidase during storage (Wu et al., 2013). Storage of shredded carrots at low temperatures can heal, inducing a positive effect on the synthesis of phenolic substances and extending the shelf life of the product (Alegria et al., 2016). In our study, the reduced H_2_O_2_ content at low temperature suggested the inhibition of protective protein expression, and it was difficult to initiate the fruit system to acquire resistance in tubers (Xu and Tian, 2008). Thus, the healing effect on potato tubers is difficult to achieve under low temperature storage.

A variety of metabolism was involved in the formation of tuber wounds, among which, sugar metabolism, phenylpropanoid metabolism, and fatty acid biosynthesis played an important role in the formation of wounds (Schmutz et al, 1996; Graca and Pereira, 2000a; Su et al., 2021). We found that low-temperature treatment suppressed sugar metabolism in tubers, which may inhibit the supply of energy and precursor substance during healing (Su et al., 2021). *StAMY23* and *StBAM1* play distinct roles in starch degradation, particularly under cold conditions. From our results, the expression level of *StAMY23* was lower at low temperatures during healing, and reducing the soluble sugar content (**Figures 1G, 2**). Silencing *StAMY23* resulted in a lower accumulation of soluble sugar in potato tubers stored at 4°C (Hou et al., 2017). Different from *StAMY23*, the *StBAM1* response was induced at low temperatures during healing (**Figures 2C**). Previous studies have demonstrated that both the expression of *StBAM1* and *StINV1* were constantly induced by low temperatures (Liu et al., 2021). The expression of *StINV1* in the low-temperature treated potatoes was up-regulated, accelerating sucrose catabolism and decreasing sucrose content (Liu et al., 2021). In addition, the expression of *StAGPase* was up-regulated and promoted starch synthesis (**Figure 1G, 2**). The starch content of common wheat can be increased by overexpression of *AGPase* (Kang et al., 2013). Nakatani and Komeichi (1992) reported a positive correlation between AGPase activity and the starch content in the tubers of sweet potatoes. Thus, the low temperature inhibited the degradation of starch under the combined effect of *StAMY23* and *StAGPase*, reducing the required energy substance. It is well known that long-term storage of potatoes at low temperatures results in cold-induced sweetening (CIS) (Xie et al., 2018). In this study, sucrose and soluble sugar contents were elevated in low-temperature treatment tubers at a later stage, which was presumably attributed to the sustained cold response of *StBAM1* and *StINV1*. The expression of genes related to sugar metabolism is variable at low temperatures. There are still no studies demonstrating the differential expression of genes related to glucose metabolism in the wound periderm tissue and inside the tuber during the later stages of healing, and it is worth investigating.

Phenylpropanoid metabolism plays a vital role in plant defense reactions (HYODO, 1976; Ferrer et al., 2008). PAL and 4CL directly regulate the process of the synthesis of phenolics, particularly wound-induced metabolism (Li et al., 2022). Our study found the expression of *StPAL1* was continuously inhibited by low temperatures, thus suppressing the conversion of phenylalanine to cinnamic acid and other phenolic acid monomers. The expression of *St4CL* was consistent with the trend of *StPAL1* expression. Zhang et al. (2021) showed that up-regulation of PAL and 4CL activity and corresponding gene expression resulted in higher metabolite production in goji berry. Similar results have been reported in peach fruit (Zhou et al., 2020). Furthermore, *StPrx* involves in the crosslinking of phenolics and promotes the formation of a closed layer around the wound subcutaneously (Espelie and Kolattukudy, 1985). The expression of *StPrx* has been maintained at a high level during the formation of the tuber seal layer (Lulai and Neubauer, 2014). However, expression of *StPrx* in low temperature treated tubers was down-regulated and remained stable. It can be explained that this gene expression is delayed and therefore not yet involved in subsequent wounded periderm formation. Overall, these results demonstrated that phenylpropanoid metabolism was inhibited, reducing synthesis of lignin and phenolics, resulting in delayed SPP biosynthesis at low-temperature in potato tubers.

Fatty acids can synthesize and deposit the biopolymer suberin at the wound site. Suberin provides protection from dehydration and pathogens (Bajji et al., 2007). Fatty acids produce fatty acyl CoA through the action of long-chain acyl CoA synthase, which is converted into super long-chain fatty acid CoA under the action of KCS (Cassagne et al., 1994). According to our study, *StKCS6* was lowly expressed in the periderm of potato tubers under low-temperature treatment (**Figure 3B**), which suppressed subsequent SPA assembling. It was shown that silencing the *StKCS6* in potato tuber led to decreased periderms, suggesting that *StKCS6* deficiency affects SPA deposition (Serra et al., 2009). Similarly, the expression of *StFAOH* and *StGPAT5* was kept at a low base level in low-temperature treated tubers (**Figures 3C, D**), which was similar to previous reports (Lulai and Neubauer, 2014). These results illustrate that the fatty acid biosynthesis was suppressed at low temperatures, resulting in difficulty in the deposition of SPA at the wound site of the tubers.

## Data Availability

The original contributions presented in the study are included in the article/**Supplementary Material**. Further inquiries can be directed to the corresponding authors.

## 5 Conclusion

Low-temperature inhibited the sugar metabolism-related gene *StAMY23*, alleviating starch degradation, and making it difficult for tubers to provide the energy required during healing. Meanwhile, the reduced H_2_O_2_ content at a low temperature has a negative effect on the synthesis of phenolics, and the reduced level of *StPrx* expression inhibited the deposition of SPP in the wound site of tubers. Furthermore, low-temperature treatment inhibited fatty acid biosynthesis-related genes *StKCS6*, *StFAOH* and *StGPAT*, thus, decreasing the SPA deposition of potato tubers during healing. Expression levels of *StPAL1* and *St4CL*, which are related to phenylpropanoid metabolism, were also inhibited by low-temperature treatment to varying degrees, suppressing the formation of lignin and phenolics during healing. This study elucidated that low-temperature treatment reduces potato wound formation by inhibiting phenylpropanoid metabolism and fatty acid biosynthesis.
