# Supplementary material for: Low temperature reduces potato wound formation by inhibiting phenylpropanoid metabolism and fatty acid biosynthesis
Source: Front Plant Sci. 2023 Jan 18;13:1109953. doi: 10.3389/fpls.2022.1109953 (PMC9889875; doi:10.3389/fpls.2022.1109953)
Supplement: Supplementary file 1 [file Table_1.docx]

Supplementary Table1: Gene information and primer sequences used for RT-qPCR analyses

| **Gene Name** | **Gene ID** | **Forward Primer** | **Reverse Primer** |
| --- | --- | --- | --- |
| EF1α | LOC102577640 | ATTGGAAACGGATATGCTCCA | TCCTTACCTGAACGCCTGTCA |
| StPAL1 | LOC102582618 | TGCTCCTCTTCCACTTCG | CCGTCTCAAATCCTCCAA |
| St4CL | LOC102596056 | GTGTTTGCGTTTATTGGC | GCGTAGTCCTTCACTTTCC |
| StPrx | LOC125848031 | CAGCAACCAAGGTATAATGTTT | CGCGGATGGAGGCAAGTCT |
| StKCS6 | LOC102603922 | AACCGCACAATCAAGACACCA | TCTCTGGATGAACACTGGGT |
| StFAOH | LOC102577568 | AGGCTTTGGATTCCGCGTAAG | CGACAGATGATGTGTCACGT |
| StGPAT5 | LOC102604721 | CTAGAGGATGGAAAGGACTG | GTCCCAGAATTTTGTGACAC |
| StAMY23 | LOC102577485 | GGCATACACAGCCGTTCATCT | ATCCGTCCCCAATCTTCACG |
| StBAM1 | LOC102584887 | TGAGATGCGTGACCATGAGC | CAAGTGGAACTTGCGCTTCC |
| StAGPase | LOC102577790 | GGAGTCCGATTCAATGTGAGAAGAAG | CCAAAACACTCCGGCTAGCATC |
| StINV1 | LOC102587290 | CAGGGTCTAGCGTGACTGC | TGATGGGACATCGGTGAAA |
